# Unmasking a Rare Genetic Puzzle: Hereditary Hemorrhagic Telangiectasia in a Black Kenyan Woman: A Case Report

**DOI:** 10.1155/crgm/3692365

**Published:** 2026-02-23

**Authors:** Lavender Otom, Priyanka Panwar, Farida Kaittany, Ben Clement Lomatayo

**Affiliations:** ^1^ Department of Internal Medicine, Jaramogi Oginga Odinga Teaching and Referral Hospital, Kisumu, Kenya; ^2^ Department of Internal Medicine, The Aga Khan Hospital, Kisumu, Kenya, agakhanhospitals.org

**Keywords:** case report, diagnostic challenges in HHT, epistaxis, gastrointestinal bleeding, hereditary hemorrhagic telangiectasia, resource-limited settings, systemic therapy for HHT, tacrolimus in HHT

## Abstract

**Background:**

Hereditary hemorrhagic telangiectasia (HHT) is a rare genetic disorder characterized by mucocutaneous and visceral telangiectasias, often leading to severe complications. This case report presents an uncommon manifestation of HHT in a 57‐year‐old Black Kenyan female with upper gastrointestinal bleeding. Given the rarity of HHT in our region, this case underscores the importance of early recognition, particularly in resource‐limited settings, to improve patient outcomes.

**Case Presentation:**

A 57‐year‐old Black Kenyan female presented with recurrent upper gastrointestinal bleeding. Endoscopy revealed multiple telangiectatic lesions in the stomach and duodenum, with an actively bleeding duodenal telangiectasia. Despite limited diagnostic resources, a thorough history and focused clinical examination led to the diagnosis of HHT. She was managed with blood transfusions, intravenous iron, tranexamic acid, and supportive therapy to control bleeding. Systemic therapy with low‐dose tacrolimus was later initiated for recurrent gastrointestinal bleeding. This case illustrates the diagnostic and therapeutic challenges faced in a low‐resource setting.

**Conclusion:**

This is the first documented case of HHT with upper gastrointestinal bleeding reported in Western Kenya. Raising awareness of this rare condition among healthcare providers can facilitate early diagnosis and intervention, ultimately improving patient outcomes.

## 1. Background

Hereditary hemorrhagic telangiectasia (HHT), commonly known as Osler–Weber–Rendu syndrome, is an uncommon chronic condition with a substantial impact on quality of life [1, 2]. This disorder is marked by multisystemic vascular malformation affecting the skin, mucous membranes, and various internal organs [3, 4]. The vascular abnormalities in HHT manifest as arteriovenous shunts, which are abnormal pathways linking arteries and veins directly [2, 5]. Smaller lesions are termed as telangiectasia, while larger ones are classified as arteriovenous malformations (AVMs) [2].

While HHT is well‐recognized in clinical practice, it remains underdiagnosed, particularly in resource‐limited settings where access to specialized care and diagnostic tools may be limited. The condition’s clinical presentation can be highly variable, often delaying diagnosis and treatment. This case highlights the challenges in diagnosing and managing HHT in a resource‐constrained environment and underscores the importance of early recognition to improve patient outcomes.

## 2. Case Presentation

This report highlights a 57‐year‐old Black Kenyan woman with a longstanding condition of frequent nosebleeds that began at the age of 17. She had been under follow‐up at an ear, nose, and throat (ENT) clinic and underwent multiple sessions of electrocautery, which eventually led to septal perforation. However, there was no documented ENT evaluation available to confirm the pattern, size, or extent of the perforation. This information was based solely on the patient’s recollection during history taking (an ENT review was not sought during her presentation to our facility, limiting further assessment of this complication). Despite years of follow‐up, a definitive diagnosis for her symptoms was not established.

She presented to our facility with a 6‐month history of recurrent hematemesis and melena stool, accompanied by severe anemia requiring blood transfusion on two occasions. She reported fatigue, palpitations, presyncope, and dyspnea on mild exertion. Notably, there was no antecedent epistaxis prior to the bouts of hematemesis, thus making swallowed blood an unlikely cause of her gastrointestinal symptoms.

**FIGURE 1:**
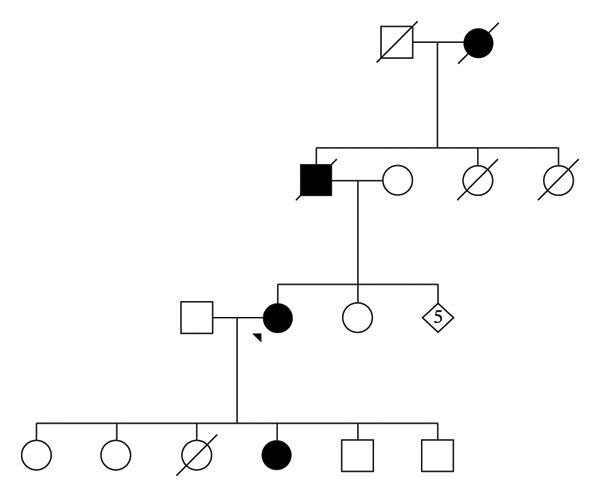
Genogram.

The patient also had a positive family history of frequent nosebleeds affecting her paternal grandmother, father, and one of her daughters (Figure 1). She was para 6 + 0 with five living children, delivered via spontaneous vertex delivery without complications, although her epistaxis episodes worsened significantly during pregnancy.

**FIGURE 2:**
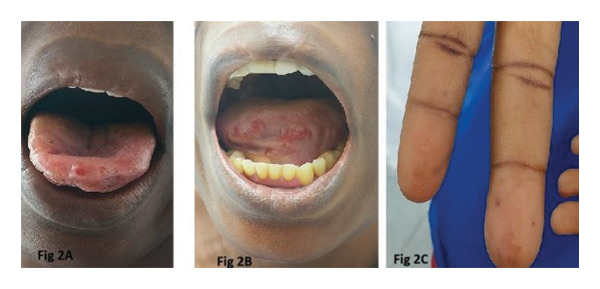
(A) and (B) oral telangiectasia; (C) palmar telangiectasia.

On examination, she appeared severely pale, with multiple pink‐red punctate lesions on the oral mucosa, tongue, and palms that blanched on pressure (Figure 2). Cardiovascular examination revealed a bounding pulse, tachycardia, and a hemic murmur. Abdominal examination identified an enlarged, nontender liver with a span of 14 cm and a tipped spleen, but no venous hums or bruits were auscultated. Other systemic findings were unremarkable. There were no stigmata of dysembryogenesis noted on examination.

Laboratory results revealed severe anemia, with hemoglobin measured at 3.1 g/dL, mean corpuscular volume of 61.6 fL, platelets of 193 × 10^9^/L, white blood cell count of 3.76 × 10^9^/L, ferritin of 1 ng/mL, and normal renal function, liver function, thyroid function, and coagulation profiles.

Based on her clinical presentation, HHT was diagnosed, meeting three of the four Curaçao Criteria, which classifies this as definite HHT. Differential diagnoses such as hereditary coagulopathies, including von Willebrand’s disease, were ruled out based on her normal coagulation profile. Although she had three first‐degree relatives with a history of recurrent nosebleeds, they had not been formally evaluated for HHT lesions and were therefore categorized as possible HHT.

**FIGURE 3:**
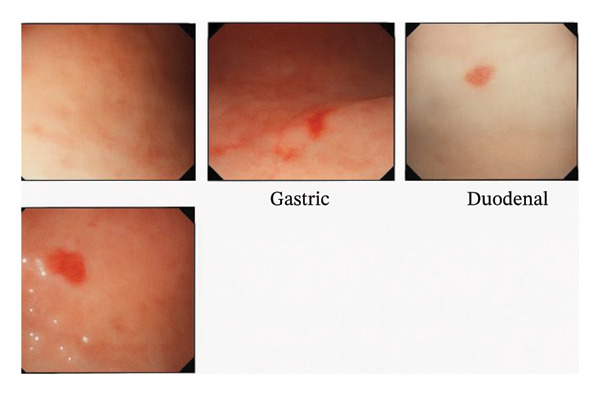
Telangiectasia noted on the gastric and duodenal mucosa.

The patient was admitted and transfused four units of packed red cells, with her hemoglobin improving to 8.6 g/dL. She also received intravenous ferrous carboxymaltose 500 mg and underwent oesophagogastroduodenoscopy (OGD). During the procedure, she experienced acute large‐volume epistaxis (∼300 mLs). Telangiectasias were identified in the stomach and duodenum (Figure 3), with active bleeding noted from a duodenal lesion during scope withdrawal. Contrast‐enhanced CT scans of the chest and abdomen revealed no pulmonary or hepatic AVMs; however, a smooth hepatomegaly was demonstrated.

She was stabilized with intravenous tranexamic acid and received an additional unit of blood. A long‐term nasal saline spray regimen was initiated, to be administered twice daily. The patient was advised on referral to a tertiary center for Argon Plasma Coagulation, but due to financial constraints, this option was not feasible 3 months postdischarge.

For the first 3 months after discharge, she remained relatively stable. However, she subsequently began experiencing episodes of significant hematochezia, leading to a drop in hemoglobin levels to 4 g/dL. The patient was readmitted for further blood transfusions and intravenous tranexamic acid. A colonoscopy was deferred due to financial limitations. During this admission, treatment options for systemic therapy were discussed, considering the possibility of colonic telangiectasia. Bevacizumab therapy and low‐dose tacrolimus were both considered.

After a comprehensive discussion weighing the benefits and risks of each treatment option, a shared decision was made to initiate low‐dose tacrolimus therapy. At her 6‐week post‐discharge follow‐up, the patient showed signs of improvement. She had been free of melena and hematochezia for 4 weeks since starting tacrolimus. Although she experienced mild gastrointestinal side effects, they were well tolerated. She continues with monthly follow‐up visits, with the goal of stabilizing her hemoglobin levels while on systemic therapy.

Financial and logistical constraints limited her access to additional genetic evaluation. She was encouraged to bring her daughter for evaluation and management, and genetic testing for her children was discussed, though also constrained by availability and cost.

This case underscores the delayed diagnosis of HHT, attributable to factors such as low clinical suspicion due to the rarity of the condition, limited access to tertiary healthcare, and the absence of advanced diagnostic tools in resource‐limited settings. It highlights the importance of recognizing this rare disorder to enable earlier diagnosis and improve patient outcomes.

## 3. Discussion

HHT affects individuals across all ages and backgrounds, with an estimated prevalence of 1:5000 [6]. So far, only two cases of HHT have been documented in Kenya [1, 7]. HHT is an autosomal dominant disorder with variable penetrance and expression [4, 8]. Approximately 90% of cases are attributed to heterozygous mutation of the endoglin (*ENG*) or the activin‐like receptor kinase 1 (*ALK1 or ACVRL1*) genes, corresponding to HHT types 1 and 2, respectively [4]. Mutations in *SMAD4*, associated with juvenile polyposis, account for fewer cases, alongside rarer associations with *GDF2*, *BMPR2*, and *RASA-1*, and loci such as 5q31.3, 5q32, and 7p14 [4, 6, 8]. Emerging evidence suggests the potential discovery of additional HHT‐associated genes in the future.

The *ENG* gene encodes a vascular endothelial glycoprotein and a part of the TGF‐β‐BMP receptor complex, which binds to TGF‐β1 and TGF‐β2 with high affinity. Similarly, *ACVRL1* encodes a cell surface receptor for TGF‐β superfamily ligands, while *SMAD4* functions as an intracellular mediator in the TGFβ‐BMP pathway [5]. The TGF‐β signaling pathway is crucial for endothelial cell proliferation, differentiation, adhesion, and migration, processes integral to angiogenesis [4, 5]. In HHT, vascular walls are thin, narrow, and tortuous and located near the surface of the skin or mucous membranes, lacking contractile properties, which predisposes to spontaneous and uncontrolled hemorrhage [5].

HHT manifestations vary depending on the affected organ. By age 16, 71% of individuals exhibit symptoms, increasing to over 90% by age 40 [8]. The most common presentation is epistaxis, affecting up to 95% of patients, with onset often before age 20 and almost universally by age 45 [2, 9]. Episodes range from mild daily occurrences to severe, sporadic nosebleeds that can lead to iron‐deficiency anemia [5, 9, 10]. Our patient exhibited this typical presentation.

Approximately 80% of individuals with HHT exhibit telangiectasias in the stomach or small intestine, which can be detected via endoscopy or capsule endoscopy [11]. GI bleeding, while less common, affects about a quarter of adults with HHT, with most cases presenting after the age of 50 [5]. Notably, this presentation shows a female preponderance in a ratio of 2‐3:1 [12]. Our patient presented with overt GI bleeding at the age of 57. However, asymptomatic chronic intermittent bleeding without notable melena is a more frequent pattern, often delaying diagnosis. Telangiectasias in the stomach and duodenum are more frequently involved than those in the colon and are major contributors to overall bleeding [13]. A thorough evaluation, ideally including both capsule endoscopy and colonoscopy, could have helped identify lesions in additional locations. However, financial constraints made this unfeasible for our patient. In HHT, endoscopic evaluation is particularly valuable in patients presenting with anemia or iron deficiency, as was the case in this presentation.

Other GI manifestations of HHT include hepatic AVMs, hepatomegaly, cirrhosis, hepatic angiomas, jaundice, portal hypertension, hepatic encephalopathy, biliary necrosis, and ascites. These occur in 41%–74% of those affected, with the average age of presentation being 52 [7].

Hepatic involvement in HHT spans a broad spectrum, from small AVMs to large confluent vascular masses, and may include arterio‐systemic shunts between the hepatic artery and hepatic vein, as well as arterio‐portal and portosystemic shunts [10]. Marked enlargement of the hepatic arteries can serve as a key indicator of shunting, which may alter hepatic perfusion and predispose to focal nodular hyperplasia or regenerative nodular hyperplasia [6]. Diagnostic evaluation typically involves Doppler ultrasound, multiphasic contrast‐enhanced CT scans, or abdominal MRI with contrast to identify and characterize these lesions [6, 10]. Careful interpretation of contrast‐enhanced imaging can help distinguish true vascular masses from FNH‐like lesions and identify potential complications such as bilomas. When hepatic lesions remain indeterminate on CT, advanced MRI sequences or liver‐specific contrast agents may improve diagnostic accuracy, particularly in differentiating hepatocellular from nonhepatocellular lesions [14]. In cases where hepatomegaly is present, as noted in the case described by Kiyeng et al. screening for liver AVMs is strongly indicated to guide management and prevent complications [1, 6]. In our patient, a screening CT scan confirmed hepatomegaly but revealed no hepatic AVMs, and further evaluation was not pursued due to financial constraints.

Pulmonary AVMS (pAVMs) are reported in 15%‐50% of individuals with HHT and are typically diagnosed by age 43 [15]. Complications arising from pAVMs include cerebral abscesses, strokes, transient ischemic attacks, massive hemoptysis from rupture, spontaneous hemothorax, and paradoxical emboli [8, 15–18]. These risks underscore the guideline recommendation to screen all HHT patients for pAVMs at diagnosis using transthoracic contrast echocardiography. Positive findings should be verified using unenhanced multidetector thoracic CT with thin slices [6]. Once diagnosed, pAVMs are treated with transcatheter embolotherapy, targeting feeding artery diameters of ≥ 3 mm, though treatment of smaller diameters (≥ 2 mm) may also be appropriate [10, 19]. Long‐term management for patients with pAVMs includes antibiotic prophylaxis before procedures that carry a risk for bacteremia, careful handling of intravenous accesses to prevent air embolism, and avoidance of scuba diving due to the risk of air embolism [10].

Although our patient did not experience HHT‐related complications during pregnancy, untreated pAVMs pose a significant risk of hemorrhage and stroke. Embolization of pAVMs during pregnancy is recommended starting in the second trimester unless otherwise contraindicated [5, 6]. Patients with pAVMs can safely receive spinal anesthesia without prior screening for spinal HHT. While vaginal delivery is generally not contraindicated, caution is advised for patients with high blood pressure or high‐risk cerebral AVMs [6]. In such cases, a cesarean section or an epidural with assisted second‐stage labor should be considered [6, 20, 21].

Cerebral AVMs (cAVMs) and spinal AVMs are reported in approximately 23% of patients with HHT [22]. While cAVMs are typically congenital, pulmonary and liver vascular malformations tend to develop over time [5]. The annual bleeding risk associated with cAVMs in HHT is estimated to be 0.5% per year [23]. Testing for cAVMs is advised for all adults with possible or definite HHT. MRI with gadolinium contrast remains the most sensitive diagnostic tool for detecting cAVMs [10]. Identifying these lesions is critical, as it allows for timely intervention in specialized centers equipped with neurovascular expertise [5, 10, 24].

HHT is diagnosed based on the Curaçao Criteria, a well‐established set of clinical guidelines. These criteria include four key components: spontaneous recurrent epistaxis, the presence of telangiectasias at multiple sites, such as the lips, oral cavity, fingers, and nose; visceral lesions, including GI telangiectasias; and a family history, specifically having a first‐degree relative with confirmed HHT or a genetic diagnosis of condition. A diagnosis is classified as ‘*definite’* if three or more of these criteria are met, ‘*possible’* or *‘suspected’* if two criteria are present and ‘*unlikely’* if fewer than two criteria are met [25]. While the Curaçao Criteria remains the traditional diagnostic tool, genetic testing is an increasingly important diagnostic method, as it allows for the identification of specific HHT mutations within families. This can facilitate early diagnosis, particularly for children and young adults who might not yet meet the Curaçao Criteria [10]. However, in resource‐limited settings like ours, where genetic studies are often inaccessible, the application of the Curaçao Criteria remains pivotal for diagnosing HHT at both primary and tertiary health centers. In our case, the patient met three of the criteria, resulting in a ‘*definite’* diagnosis of HHT. Similarly, both previously reported cases in our setup were classified as ‘*definite’* HHT based on these criteria [1, 7].

The management of the two key presentations in our case, namely, epistaxis and GI bleeding will, be highlighted below.

In managing epistaxis, conservative first‐line approaches focus on moisturizing and humidifying the nasal mucosa to prevent dryness and crusting [6]. Nasal saline sprays and thermosensitive gels, applied twice daily, are effective in reducing the frequency of nosebleeds [26–28]. In acute episodes, nasal packing is often used, but strategies like lubricated low‐pressure pneumatic packing or lubricated packing are preferred, as they reduce the risk of rebleeding upon removal [6]. When these initial strategies fail, oral tranexamic acid, which has minimal adverse effects, may be employed [6, 29]. Our patient had previously received some of these therapies, though nasal sprays were sparingly used after episodes of epistaxis had resolved. Notably, a 2024 trial demonstrated that pomalidomide therapy notably decreased epistaxis severity scores in individuals with moderate to severe epistaxis over a 24‐week period, highlighting a potential future addition to treatment guidelines [30]. Ablative therapies, including laser treatment, radiofrequency ablation, electrosurgery, and sclerotherapy, are used for nasal telangiectasias but yield variable results and are associated with documented risks [6, 31–33]. In refractory cases, systemic antiangiogenic agents like bevacizumab, thalidomide, septodermoplasty, or nasal closure procedures such as Young’s procedure can be considered [6, 32, 34, 35]. Additional interventions like arterial ligation, electrocautery, and sprayable fibrin sealants are also options, though electrocautery is not typically a first‐line choice [32, 36]. In our resource‐limited setting, electrocautery was the readily available treatment option, consistent with findings previously reported by Kitonyi et al.

In managing anemia in patients with HHT, the initial approach typically involves oral iron supplementation, which is often sufficient to correct iron deficiency. However, if oral iron therapy proves ineffective or poorly tolerated, IV iron therapy may be considered as an alternative treatment. In more severe cases, such as those with significant blood loss or ongoing bleeding, blood transfusions may be necessary [6]. Transfusions are particularly indicated for patients experiencing shock, those with underlying health conditions that necessitate a higher hemoglobin level, or in cases requiring acute increases in hemoglobin, such as during pregnancy or surgery [6, 37, 38].

Gastrointestinal bleeds in HHT are classified as mild, moderate, or severe. Mild cases can be managed with oral iron, while moderate cases require intravenous iron, and severe cases may necessitate blood transfusion [6]. Oral antifibrinolytic therapy can be administered in individuals with mild HHT‐related GI bleeding, and systemic antiangiogenic therapies, such as bevacizumab, thalidomide, and lenalidomide, are indicated in moderate to severe cases [6, 39, 40]. Low‐dose tacrolimus has also shown promising results. Endoscopic argon plasma coagulation ought to be used judiciously during OGD, particularly during the initial endoscopic evaluation of bleeding lesions or significant nonbleeding lesions (1–3 mm) [41]. Repeated sessions are discouraged due to the risk of iatrogenic intestinal injury, including bowel perforation and strictures [6, 42]. Other useful therapies include estrogen‐progesterone combinations, tamoxifen, and raloxifene, which can be considered for postmenopausal women with HHT‐related GI bleeding. Nonetheless, the potential advantages and disadvantages of these treatments need to be thoroughly evaluated prior to administration [36]. In the case of our patient, low‐dose tacrolimus therapy was elected after a comprehensive discussion, with the decision considering benefit, cost/availability, and risks. She was counselled on the fact that this was not a guideline‐recommended first‐line therapy and that if treatment failure was noted upon follow‐up, then we would consider the antiangiogenic agent bevacizumab that is guideline‐recommended.

### 3.1. Prognosis

With optimal management, including epistaxis control, treatment of anemia, and monitoring and treatment for pAVMs, patients with HHT can achieve a life expectancy comparable to that of the general public. Interestingly, modulation of angiogenesis in HHT patients is associated with a protective effect against certain cancers [43]. Conversely, in settings where patients are not optimally managed, life expectancy may be modestly reduced, with one study reporting a reduction of approximately 3 years [44].

### 3.2. Strengths

• Comprehensive Exploration: The case report thoroughly addresses the pathophysiology, clinical manifestations, and diagnostic and management approaches for HHT.
• Timely Diagnosis: The prompt diagnosis of HHT at presentation facilitated the identification of gastrointestinal mucosal lesions and enabled the consideration of systemic therapy.
• Research Insight: The report entails a trial of low‐dose tacrolimus therapy and will provide valuable insight into the clinical response to this understudied drug therapy.

### 3.3. Weaknesses

• Absence of Genetic Screening: The lack of genetic evaluation to confirm the causative mutation for HHT is a notable limitation. This restricts the ability to diagnose asymptomatic relatives, potentially leaving high‐risk individuals unidentified.
• Lack of Long‐Term Follow‐up: Although the patient showed a positive response to the initial low‐dose tacrolimus therapy, the 6‐week follow‐up period was too short to fully assess improvements in hemoglobin levels or to evaluate potential adverse effects of the treatment.
• Single Patient Focus: As a single case report, its findings may have limited generalizability. Including additional cases with similar presentations could improve external validity and enhance understanding of HHT within this clinical context.

## 4. Conclusion

This case report highlights a rare medical condition, emphasizing the importance of focused history‐taking, targeted clinical examination, and appropriate investigations. It also draws attention to significant challenges in resource‐limited settings, such as restricted access to advanced diagnostic tools and therapies. The absence of genetic testing was a critical barrier to diagnosing HHT among asymptomatic relatives, potentially impacting early identification and management. Furthermore, the absence of long‐term monitoring restricts the evaluation of the effectiveness of the interventions. Despite these limitations, the report underscores the need for heightened clinical suspicion and comprehensive care to improve outcomes for patients with HHT.

**Nomenclature**
HHT: Hereditary hemorrhagic telangiectasia
GI: Gastrointestinal
AVMs: Arteriovenous malformations
OGD: Oesophagogastroduodenoscopy
ENG: Endoglin
ACVRL1: Activing‐like receptor kinase 1
pAVMs: Pulmonary arteriovenous malformations
cAVMs: Cerebral arteriovenous malformations
IV: Intravenous

## Author Contributions

Farida Kaittany performed the endoscopy and played a key role in patient follow‐up. Lavender Otom and Priyanka Panwar were responsible for drafting and writing the manuscript. Ben Clement Lomatayo critically reviewed the case report.

## Funding

The authors received no specific funding for this work.

## Ethics Statement

The authors have nothing to report.

## Consent

Written informed consent was obtained from the patient for the publication of this case report and any accompanying images. All identifying details have been removed to protect the patient’s privacy.

## Conflicts of Interest

The authors declare no conflicts of interest.

## Data Availability

All data supporting the findings of this case report, including clinical details and images, are fully included within the published article. No additional data are available.
